# Secretion of Milk, Excited by Nursing, in the Case of a Grand-Mother

**Published:** 1855-10

**Authors:** J. Boring

**Affiliations:** Professor of Obstetrics, &c., Atlanta, Georgia


Article II.—*Secretion of Milk, excited by nursing, in the case of a Grandmother.* By J. Boring, M. D., Professor of Obstetrics, &c., Atlanta, Georgia.

Well attested instances of the secretion of milk by unimpregnated and non-childbearing females, as also of males, abound in most of the standard works of the medical profession, and therefore require no accession of facts for their confirmation. Hippocrates, Baudelocque, Gordon Smith, Sir Hans Sloane, Horner, Dr. Francis, of New York, and others, have contributed to demonstrate the fact.

"Baudelocque mentions the case of a girl, eight years old, who milked her breasts in the presence of the Royal Academy of Surgery October 16, 1783, and Belloc another; in both the secretion was apparently the result of the application of a child to the breasts.

A similar case, but in a woman, is related by Wm. Semple in the north of England. Medical and Surgical Journal, vol. I, p. 230." *Churchill's System of Midwifery.*

Sir Hans Sloane gives the case of a lady who, at the age of sixty-eight years, not having borne a child for more than twenty years, nursed her grand children one after another.

Dr. Francis, of New York, mentions a lady who, fourteen years subsequent to her last labor, continued a copious secretion of milk, and who might constantly have performed the duties of a wet nurse.

Other instances of similar character, equally well established, are to be found, but these are sufficient for my purpose.

A little more remarkable is the fact that *males* have been known to assume and perform this interesting function. The Bishop of Cork, 1791, gives the case of a man who suckled his child after the death of his wife. Humboldt and Captain Franklin have given similar cases.

Dr. Horner, in a note to his work on Anatomy, relates the case of a *male* patient, in the Philadelphia Alms House, in whom the breasts were as large as those of a nursing woman, though the nipples were not well developed. His voice was effeminate, and the genital organs about the size of those of a boy fifteen years old.

But the most remarkable and interesting case of the kind, of which we have any knowledge, is recorded by Dr. Dunglinson in his work on "Human Physiology." Vol. 2d., p. 439, 4th Ed. He says: "Professor Hall, of the University of Maryland, exhibited to his obstetrical class in the year 1837 a colored man, fifty-nine years of age, who had large, soft, well-formed mammæ, rather more conical than those of the female, and projecting fully seven inches from the chest, with perfect and large nipples. The glandular structure seemed to the touch to be exactly like that of the female. This man had officiated as wet nurse for several years in the family of his mistress, and he represented that the secretion of milk was induced by applying the children intrusted to his care to the breasts during the night. When the milk was no longer required, great difficulty was experienced in arresting the secretion. His genital organs were fully developed."

To the foregoing cases, I propose adding that of Mrs. J., of this city, whose history in this respect has recently come to my knowledge.

On the 15th of July last I was called to see a child suffering with diarrhœa, at the house of the husband of this lady, and in the course of my visitations obtained the following facts:

Mrs. J. is now about forty-seven years of age, rather thin, frail, and of nervous temperament. She was married at the age of twenty-three; has borne three children, and suffered one abortion. She nursed her last child thirteen years since, and until recently has not secreted a vestige of milk.

In April last her only living child, a married daughter, gave birth to her first born and died a few days afterwards, leaving the child to her mother, (Mrs. J.,) who, to soothe it at night and when sick, applied it to the breasts. This practice was continued up to the time when I saw the parties, at which, and to the last of my knowledge of them, there was an abundant secretion of milk. This lady is the Grandmother of the child she nurses, and has not suckled a child for the space of thirteen years.

I should have stated that regularly recurring menstruation has continued in the case of Mrs. J. since the birth of her child, thirteen years since, and no material change has occurred in her general health.

In presenting this subject, and the enumeration of the foregoing facts, it is not my purpose to seek the establishment of any new physiological doctrine. The cases given, and experiments had, bearing on this subject, are too few and uncertain to warrant a satisfactory conclusion. I consider, however, that the following propositions have long since been put beyond controversy, viz:

1. That the chaste and unimpregnated *virgin* may be the subject of enlargement of the mammary glands and the secretion of milk, which, while it is of great interest to the physician, constitutes an important principle, involved in medical jurisprudence.

2. That the secretion of milk may occur in women who are incapacitated by age for child-bearing, which also involves numerous points of importance to the medico-jurist.

3. That the presence of the uterus is not indispensable to the performance of the function of lactation, there being numerous instances, well-established, of its exercise in *males*.

4. That the mammary gland in both sexes is the same, except that in the male it is in *miniature; the structure is the same.*

To this, Horner, Carpenter, Dunglinson, and &c., all agree.

